# Prey movement, size, and glossiness interact to impact praying mantid attack behaviors

**DOI:** 10.1093/beheco/araf107

**Published:** 2025-09-10

**Authors:** Yvonne Wang, Devi Stuart-Fox, Patricia Henríquez-Piskulich, Amanda M Franklin

**Affiliations:** School of BioSciences, The University of Melbourne, Parkville, VIC 3010, Australia; School of BioSciences, The University of Melbourne, Parkville, VIC 3010, Australia; School of BioSciences, The University of Melbourne, Parkville, VIC 3010, Australia; School of BioSciences, The University of Melbourne, Parkville, VIC 3010, Australia; Department of Ecological, Plant and Animal Sciences, La Trobe University, Bundoora, VIC 3086, Australia

**Keywords:** anti-predator coloration, body size, glossiness, protean movement, specularity

## Abstract

When moving, animals are vulnerable to predation because movement can rapidly attract the attention of a predator. To reduce the risk of predation while moving, animals can use a variety of different strategies (eg erratic movement, coloration). These strategies often work in combination or may be influenced by other prey characteristics (eg size), but few studies have explored these interactions. Here, we investigate how movement trajectory (linear or erratic), prey size (small or large) and prey coloration (glossy or matte) interact to impact the attack behavior of giant rainforest mantids (*Hierodula majuscula*). We presented mantids with animations of moving targets and filmed their response with a high-speed camera. As expected, mantids were more likely to track large than small targets and targets moving linearly than erratically. Counterintuitively, however, mantids were quicker to strike at erratically moving targets, perhaps because they more closely resembled preferred prey. When mantids attacked the target, their accuracy was influenced by the interaction of target trajectory and glossiness. As predicted, mantids had larger attack errors (ie lower accuracy) toward erratically moving glossy targets compared with linearly moving glossy targets or erratically moving matte targets. However, contrary to our prediction that linearly moving matte targets would be easiest to capture, these targets also elicited large attack errors, similar to those recorded for erratically moving glossy targets. Together, our results demonstrate that anti-predator tactics for prey in motion may interact in complex ways, and simple experimental scenarios may overlook context-dependent effects that emerge when multiple factors interact.

## Teaser

Unpredictable movement is an effective strategy for prey to evade attack from praying mantid predators. However, other prey characteristics, including size and glossiness, mediate the effect that movement trajectory has on predator behavior. Movement can rapidly attract the attention of predators and our results provide insights into how prey can evade predators while moving.

## Introduction

Balancing movement and predation risk is a critical challenge for many animals. Most animals need to move to find necessary resources, but motion cues can be used by predators to rapidly distinguish an object from the background (Regan and Beverley 1984; Rushton et al. 2007; Yin et al. 2015; Tan et al. 2024b). To reduce the risk of predation when moving, prey animals use a variety of tactics, such as motion masquerade (Cuthill et al. 2019; Tan et al. 2024b), erratic movement (Humphries and Driver 1970; Richardson et al. 2018), coloration (Scott-Samuel et al. 2023; Tan et al. 2024b), flocking (Beauchamp 2014; Hogan et al. 2017), or deceptive signaling (Golding et al. 2001; Hikidi et al. 2020). Often, prey will use multiple strategies simultaneously to improve the likelihood of survival. For example, cephalopods eject a cloud of ink to startle predators or misdirect attacks, and inking is often associated with a change in cephalopod color or movement trajectory to further confuse predators (Wood et al. 2010; Staudinger et al. 2011; Hikidi et al. 2020). To date, much of the research into anti-predator tactics during motion is theoretical or conducted with human “predators” (Tan et al. 2024b), and we lack supporting evidence from empirical studies with animal predators. Moreover, few studies have investigated how multiple strategies interact to influence prey survival, and those that do often report unexpected or conflicting results (Scott-Samuel et al. 2023; Tan et al. 2024b).

Over recent years we have started to uncover how coloration can improve survival for moving prey animals and identified that the benefit may be impacted by other prey characteristics. Several studies have provided evidence that stripes (Stevens et al. 2008; Scott-Samuel et al. 2011; Umeton et al. 2019), flashing contrasting colors (flash coloration; Murali 2018; Silvasti et al. 2024), glossiness (Henríquez-Piskulich et al. 2023; Franklin et al. 2024), and iridescence (Pike 2015) can disrupt predator tracking or localization of moving prey, increasing attack error. Some of these strategies appear to be effective against diverse predators with different visual systems, including birds (Pike 2015; Silvasti et al. 2024), praying mantids (Umeton et al. 2019; Henríquez-Piskulich et al. 2023), and jumping spiders (Franklin et al. 2024). Other factors, including prey physical characteristics and movement behavior, may interact with the prey's coloration to influence anti-predator benefits. For example, flash coloration or high contrast stripes can be more effective for fast-moving prey (Scott-Samuel et al. 2011; Umeton et al. 2019; Silvasti et al. 2024) or for prey with smaller body sizes (Murali and Kodandaramaiah 2018, 2020), and flash coloration is enhanced by erratic movement (Murali and Kodandaramaiah 2020). High glossiness is also likely more beneficial for fast-moving prey (Henríquez-Piskulich et al. 2023), but whether the benefits of a glossy appearance interact with other prey characteristics is poorly understood.

A glossy or shiny appearance is common across the animal kingdom, but only recently have we identified an anti-predator function of gloss for moving prey. Glossiness is characterized by reflecting a large proportion of incident light at the specular, or mirror, angle (Franklin and Ospina-Rozo 2021; Stuart-Fox et al. 2021). This can produce a bright flash of light and, for extremely glossy organisms, the surface reflects spectral and spatial properties of the surrounding environment (eg Denton 1971; Neville 1977; Franklin et al. 2022). Therefore, as a glossy animal moves, reflections may shift across the surface of the body, and bright flashes will be produced as the sun reflects off different body regions. If these flashes are temporally regular, such as from insect wing beats, predators may be able to use the flashes to identify and localize prey (Talley et al. 2023). However, if the flashes are temporally irregular, such as from different body regions and changes in body orientation, they may disrupt localization of prey (Franklin et al. 2024). The location, presence, and frequency of flashes will be dependent on viewing geometry, ie the position of the sun, the predator, and prey. Therefore, movement behaviors, such as erratic versus linear movement paths, could influence the appearance and frequency of these flashes and any anti-predator benefits. Prey size may also influence the effectiveness of a glossy appearance as an anti-predator strategy because as size increases, it will be easier for predators to localize and capture prey. If a glossy appearance impacts predator attack error by only a small amount, then benefits may only be experienced by smaller prey animals. Therefore, both erratic movement and size may interact with glossiness to impact anti-predator benefits for moving prey.

Praying mantids are generalist predators known to capture a wide range of flying insects, including glossy insects such as flies or beetles (Prete 1999; Kral and Prete 2004; Henderson et al. 2008; Wilson Rankin et al. 2023). Many mantid species are ambush predators and will strike at prey that move within their catch range. Numerous studies have investigated attack behaviors of praying mantids by presenting them with physical or artificial targets, providing useful information about preferred prey characteristics to elicit an attack (Prete et al. 1993, 2011, 2013; Prete and Mahaffey 1993; Nityananda et al. 2016a). Most species prefer to attack moving prey, generally 2.5 to 5 cm away, and do not have a strong size preference (Prete and Mahaffey 1993; McCue et al. 2016; Nityananda et al. 2016a). They can use both first-order motion (changes in luminance) and second-order motion (changes in non-luminance cues, such as pattern or contrast) to localize prey, and binocular stereopsis to estimate depth (Nityananda et al. 2016b, 2018, 2019). A glossy appearance may impact their perception of both first- and second-order motion, and depth perception, because gloss produces reflections off the prey animal that change with movement and these reflections are in a slightly different position for each eye (ie binocular disparity; Nityananda and Read 2017; Adams et al. 2019). Therefore, praying mantids are ideal predators to investigate the impacts of prey movement patterns and physical characteristics on prey survival and predator behavior.

Here, we investigated whether prey movement trajectory, glossiness, or size impacts predator attack behaviors and prey survival. Giant rainforest mantids (*Hierodula majuscula*) were presented with animations of moving targets, and their responses were recorded using high-speed cameras. Targets followed either an erratic or linear trajectory, were small or large, and glossy or matte. Whilst animations do not replicate all characteristics of a glossy appearance (ie binocular disparity, intensity range), they allow us to investigate if the visual changeability associated with gloss contributes to the predator's response. Erratic movement is likely to be more difficult to track and localize than linear movement; therefore, we predicted that mantids would be less likely to respond to and attack erratically moving targets than linearly moving targets (Humphries and Driver 1970; Richardson et al. 2018; Tan et al. 2024b). Similarly, small targets are likely more difficult to track and localize than large targets, and likely represent less profitable prey. As a result, we expect mantids to show low responsiveness to small targets regardless of their movement trajectory. In contrast, because large targets represent more profitable prey that is easier to capture, erratic movement may help to offset their attractiveness by increasing task complexity. Therefore, we predicted that small prey may be difficult to capture regardless of movement trajectory, whereas large prey may benefit from erratic movement. Previous research has identified that a glossy appearance can disrupt attack behaviors (Henríquez-Piskulich et al. 2023; Franklin et al. 2024) but that these benefits may only occur in scenarios where prey are more difficult to localize, such as if prey are moving quickly (Henríquez-Piskulich et al. 2023). We predicted that a glossy appearance will be more beneficial for smaller prey or erratically moving prey because these should create more difficult capture scenarios for mantids.

## Materials and methods

### Animal husbandry

We conducted experiments with 17 female giant rainforest praying mantids (*H. majuscula*) obtained from a laboratory population at the University of Melbourne. These mantids were reared from hatchlings under a 12 h:12 h light:dark cycle at 27 °C and 40% to 80% humidity. Newly hatched mantids were housed in shared mesh enclosures and fed with fruit flies (*Drosophila melanogaster*) until they reached the fourth instar. From fourth instar onward, mantids were housed individually in mesh enclosures (30 cm × 30 cm × 30 cm) and fed a live cockroach (*Nauphoeta cinerea*) 3 times per week.

Once mantids molted to adults, we began acclimating them to the experimental apparatus. For 4 wk prior to the experiment, adults were placed onto the experimental platform and then fed a cockroach. During the experiment, mantids were not fed for 8 d prior to each trial to increase motivation. We also increased the room temperature to 30 °C to increase activity levels.

### Visual stimuli

We generated animated targets with realistic glossy visual effects using Maya 2023 and the Arnold renderer plugin (Autodesk, CA, USA). Arnold is a photorealistic rendering system that uses an advanced Monte Carlo ray tracing algorithm to accurately depict lighting conditions and object surface properties. This requires specifying lighting and background conditions by uploading a 360° image of a natural background to the “aiSkydomelight” attribute. We downloaded 3 images of natural forest scenes from PolyHaven to resemble the natural habitat of mantids and produce different video replicates (Fig. S1). Because our experiment was not investigating effects of background on behavior, we specified these images to only influence target reflection characteristics and not be visible as a background. Instead, the background was set to mid-gray (red, green, blue [RGB] values: 133:133:133; Fig. 1a).

**Fig. 1.**
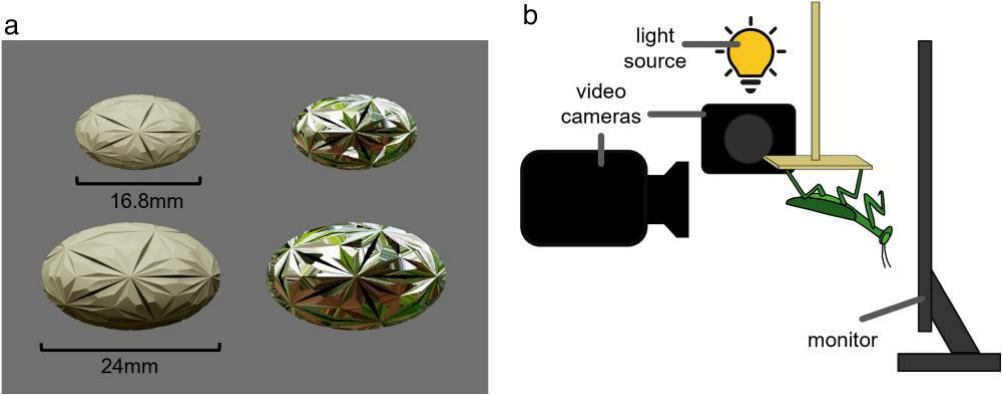
Experimental setup. a) Examples of large or small, glossy or matte targets. The color of the matte target is the average RGB values of the glossy target. b) The mantid was positioned upside-down on a platform 3 cm from the computer monitor. A high-speed camera was positioned directly behind the mantid and another at 90° to the left. A light source was positioned with the second camera to provide enough light. To minimize disturbance, the mantid and the monitor were surrounded by white corflute with holes for the camera to film through.

Four different targets were created: small glossy, small matte, large glossy, large matte (Fig. 1a). All targets were ovals with ridges and grooves. This texturing was not designed to represent a specific organism, but intended to represent that prey are not perfectly smooth so glossy reflections can unpredictably flash off different body parts during movement (eg legs, wings, body, head). Target size was selected to elicit a response from the mantid (ie not too small or too large) and to fall within the size range of prey organisms these mantids eat (eg flies, crickets, grasshoppers). Large targets were 2.4 cm × 1.44 cm (major and minor axes, respectively) with an area of 2.71 cm^2^ and small targets were 1.68 cm × 1.03 cm with an area of 1.36 cm^2^, reflecting a 50% decrease in size (Fig. 1a). Glossy targets were created using the aiStandardSurface surface attribute with metalness = 1 and roughness = 0 to create a mirror-like appearance. Matte targets were created using the aiLambert surface attribute, with the matte color set to the average RGB values of the glossy target. Therefore, the matte targets are not identical colors in the 3 video replicates because the 360° background image influences the reflectance properties of the glossy target (Fig. S2). Both glossy and matte targets are shaded underneath because light is modeled to predominantly come from the sky (ie the sun).

For all target types, we also created 2 different movement trajectories: linear and erratic. Three replicate erratic trajectories were generated using an R package “trajr,” which allows adjustments to trajectory parameters to simulate various motion paths such as directed walks, Levy walks, and Brownian motion (McLean and Skowron Volponi 2018). Insect flight behaviors vary both within and across species, often adapted to specific purposes. For example, during foraging, the dronefly *Eristalis tenax* exhibits flight patterns that mimic those of the honeybee *Apis mellifera*, characterized by frequent hovering, looping behavior and revisiting specific locations. This behavior contrasts with the more linear and direct flight paths observed in houseflies (*Musca* spp.), which typically lacks such hovering and looping during foraging (Golding et al. 2001). Here, we modeled a directed flight trajectory to replicate a scenario in which potential prey flies past a sit-and-wait predator. To create a realistic flight path, we generated 3 directed trajectories based on housefly flight data from Golding et al. (2001) (Fig. S3). We specified trajectories to consist of 300 steps, with step length sampled from a normal distribution with a mean of 1 and standard deviation of 1. Turning angles were drawn from a normal distribution with a standard deviation of *π*/2 radians (ie 90°), allowing for large directional changes. To maintain a general forward movement pattern, the turning angle for each step is added to 0 radians, rather than to the angle of the previous step. These flight paths were exported from R as PNG files, and then imported into Maya as images. Each path was manually traced within Maya using the CV curve tool, and then the target was linked to the path for its movement pattern. Linear target paths followed a horizontal linear line. Previous research found that glossiness may only impact mantid attack behaviors at fast speeds (Henríquez-Piskulich et al. 2023). Therefore, we programmed targets to move at relatively fast speed, 43 cm s^−1^, because this speed reliably elicited attack behaviors and is a moderate flight speed for insects (Dean 2003).

Animations were rendered frame by frame at 1080p resolution and played back at 75 frames per second (fps). We generated 3 replicates per treatment to incorporate variation in reflectance due to background characteristics and variation in movement trajectories, while keeping rendering demands practical. Image processing software (FIJI; Schindelin et al. 2012) was used to compile the images into MP4 videos and loop the video so the target continually moved back and forth. These animations were displayed to mantids on a Samsung (27″ S6U QHD) monitor with display dimensions of 596.736 × 335.664 mm (2,560 × 1,440 pixels) and a refresh rate of 75 Hz.

### Experimental protocol

To standardize mantid position and reduce movement, mantids were placed upside-down on a narrow platform (5 cm×8 cm) 3 cm away from the monitor (Fig. 1b). Both the monitor and the platform were surrounded by white corflute boards to minimize disturbance. These boards had 2 holes so that 2 high-speed cameras could be used to record the experiment. One camera (Photron FASTCAM NOVA Model S16 with Nikon lens 16 to 80 mm) was positioned immediately behind the mantid to capture mantid attack behaviors. This camera was set to film at 1,000 fps with 1/2,000 shutter speed. The second camera (Chronos 2.1-HD high-speed camera with a Sigma lens 105 mm 1:2.8D) was positioned at 90° to 1 side so that we could also capture strike depth information (ie if the strike hit the monitor). This camera was set to film at 1,000 fps with 1/1,000 shutter speed. To provide enough lighting for this camera, an Aputure Amaran 100x Bi-Color LED light was set behind the side camera, illuminating at 13% intensity through the camera opening. At the start of filming each day, an image of 2-mm gridded paper was recorded to allow subsequent calibration of the video footage from the rear camera.

Mantids were exposed to 2 treatment videos on each experiment day, and received a 3-d break between experiment days. Each mantid was exposed only to either small or large targets on an experimental day, as prior exposure to different sizes could affect how subsequent targets were evaluated. Presentation order of other treatments was randomized for each mantid. At the beginning of trials, a mantid was placed on the platform and allowed 5min of acclimation before the first treatment was played. Once the mantid had responded or the animation cycled 10 times (ie the target passed by 20 times, ∼2 s per pass-by), the animation was stopped and the mantid received another 5-min break before the second treatment. If a mantid did not attack the stimulus, this was noted down, and this treatment was repeated on another day, after all the other treatments had occurred (*n* = 13 mantids). Treatments were repeated up to 4 times to elicit an attack, and these repeated trials were used only to assess attack behaviors (attack latency, error, and success). Some mantids were removed from the experiment after 2 (*n* = 1) or 4 (*n* = 3) trials because they developed a black spot on their eye, which can occur during captivity. In total, we conducted 118 trials to assess the mantids’ initial responses to each treatment, and recorded 75 strikes from all trials (ie initial presentations and repeat presentations).

### Data collection

From the rear video footage, we recorded the following variables: track (yes/no), strike (yes/no), strike latency, successful strike (yes/no), and attack error. Tracking was defined as when the mantid's head or prothorax followed the target motion, and striking was when the mantid attempted to use its raptorial legs to capture the target. For these variables (ie probability of attacking or striking), analyses were only conducted on data collected from the first time the mantid was presented to each treatment. The following variables, strike latency, successful strike, and attack error, were collected for all strikes. Strike latency assessed how long mantids tracked the target before striking. It was calculated from the number of times the target passed by the mantid multiplied by the duration of 1 pass-by. While all targets were moving at the same speed, the movement path for the erratic motion is longer than for the linear motion. Linearly moving targets completed a pass in 2.00 s, while erratically moving targets took 2.24, 2.49, or 2.68 s depending on the movement trajectory replicate. A successful strike was defined as when the top spine of the tibia from either arm hit the target. Attack error was measured as the shortest distance between the closest arm and the target center during a strike; therefore, if either arm crossed the center of the target, attack error was defined as zero.

### Data analysis

To investigate the effect of glossiness, size, and trajectory on mantid hunting behaviors, we ran generalized linear mixed effect models using the glmer function (lme4 package; Bates et al. 2015) in R v4.4.0 (R Core Team 2024). For some response variables, the sample size was low relative to the number of model parameters. This is because mantids were less responsive to the treatments than expected based on pilot trials and previous experiments (Henríquez-Piskulich et al. 2023). Including all fixed effects and interactions could result in overfitting and unstable model estimates (Bolker et al. 2009; Harrell 2015). Therefore, we followed current guidelines for model comparison by defining a small set of a priori models and selecting among them using corrected Akaike information criterion (AICc) (Whittingham et al. 2006; Bolker et al. 2009; Symonds and Moussalli 2011; Harrell 2015). These models were selected to test the effect of our treatments and key interactions, but minimize the number of parameters to be estimated. This approach is preferred over step-wise model selection because step-wise model selection can result in biased estimates of parameters, standard errors and confidence intervals (CIs), and increase the type-I error rate (Whittingham et al. 2006; Harrell 2015).

We defined 5 a priori models to test the effects of glossiness, size, and trajectory and interactions between these variables on mantid behaviors (Table 1). All models included fixed effects of glossiness (matte, glossy), size (small, large), trajectory (erratic, linear), mantid experience (ie number of trials the mantid had experienced), and animation replicate. Animation replicate was included as a fixed effect rather than a random effect because random effects generally require at least 5 levels (Crawley 2002; Oberpriller et al. 2022), but animation replicate had only 3. Model 1 included all 2-way interactions between glossiness, size, and trajectory; Models 2 to 4 included only 1 of the 2-way interactions, and Model 5 included only main effects and no interactions. Each model also included a random effect of mantid ID. To identify the model that fitted the data best, we compared AICc values (model.sel, MuMIn package; Bartoń 2023). If multiple models were within 2 AIC of the top model, we took a conservative approach and reported results from the simplest model.

**Table 1.** Details of the 5 models defined a priori to investigate the effect of target glossiness, size, and trajectory on mantid hunting behaviors.

Model	Main effects	Interactions
1	Glossiness, size, trajectory, experience, replicate	Glossiness × size Glossiness × trajectory Size × trajectory
2	Glossiness, size, trajectory, experience, replicate	Glossiness × size
3	Glossiness, size, trajectory, experience, replicate	Size × trajectory
4	Glossiness, size, trajectory, experience, replicate	Glossiness × trajectory
5	Glossiness, size, trajectory, experience, replicate	None

The analyses for several of the response variables were conducted on a subset of the dataset. The probability of tracking used the data from the first time the mantid was exposed to each treatment (*n* = 118; binomial). For those mantids that tracked the target, we then investigated the effect of treatments on the probability of striking (*n* = 52; binomial). In many instances (*n* = 79), mantids did not strike at the target on the first exposure so we repeated the treatment. All subsequent analyses were conducted using instances when the mantid struck at a target, regardless of whether it was the first exposure. This includes latency until a strike (*n* = 75; Gaussian), probability of a successful strike (*n* = 75; binomial), and strike error (*n* = 61; Gaussian). For strike latency, we log-transformed the response variable to meet normality and homoscedasticity assumptions of the model. For strike error, we removed records where the mantid did not hit the screen because for these instances our error measurement was not a good reflection of whether the mantid would have captured the target (eg could be accurate in *x* and *y*, but miss the target because depth is inaccurate). Diagnostic plots (eg residual plots, QQ-plots) were used to assess model fit. To test the significance of fixed effects we ran Wald *χ*^2^ tests using the ANOVA function (car package; Fox and Weisberg 2019). The effect sizes of variables were predicted using the “emmeans” command (emmeans package; Lenth 2023).

## Results

**Fig. 2.**
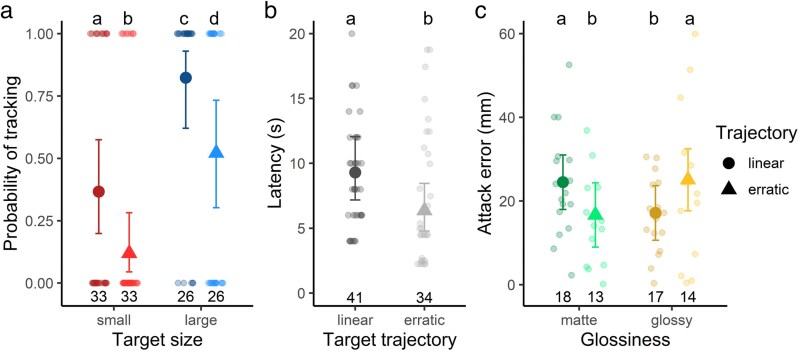
Praying mantid hunting behaviors toward targets that varied in size (small or large), trajectory (linear or erratic) and glossiness (glossy or matte). a) Probability of tracking was greater toward larger targets or those moving in a linear trajectory. b) Latency until striking was longer when presented with targets moving linearly compared with erratically. c) Attack error was larger for matte targets moving linearly and glossy targets moving erratically, and smaller for matte targets moving erratically and glossy targets moving linearly. Large point and error bars indicate maximum likelihood estimates and 95% CIs, shadowed points represent raw data, numbers on the *x*-axis represent sample sizes and letters depict significantly different groups.

The initial exposure to each treatment elicited tracking behavior in 52 trials (out of 118 trials), and the probability of tracking varied with target size (*χ* = 14.92, df = 1, *P* < 0.001; Fig. 2a; Table 2), target trajectory (*χ* = 7.63, df = 1, *P* = 0.006; Fig. 2a; Table 2), and the animation replicate (*χ* = 11.15, df = 2, *P* = 0.004). Mantids were more likely to track large targets compared with small targets, and those moving linearly compared with erratically (Fig. 2a; Table 2). Animation replicate “c” elicited tracking in only 29% of trials (12 out of 41), compared with “a” and “b” eliciting tracking in 59% and 47% of trials (a: 20 out of 34 trials; b: 20 out of 43 trials). Probability of tracking was not influenced by experience, target glossiness or interactions between the treatment variables. Furthermore, for those mantids that tracked the target, there was no impact of any variables on the probability of subsequently attacking the target (Tables 2 and 3).

**Table 2.** Maximum likelihood estimates (MLE) and 95% CI for each treatment group.

Response variable	Linear (MLE [95% CI])				Erratic (MLE [95% CI])			
	Small		Large		Small		Large	
	Matte	Glossy	Matte	Glossy	Matte	Glossy	Matte	Glossy
Probability of tracking	0.32 (0.15–0.55)	0.41 (0.21–0.65)	0.79 (0.55–0.92)	0.85 (0.64–0.95)	0.10 (0.03–0.27)	0.14 (0.05–0.34)	0.47 (0.24–0.72)	0.57 (0.32–0.79)
Probability of striking	0.83 (0.41–0.97)	0.51 (0.17–0.85)	0.97 (0.76–1.00)	0.88 (0.56–0.98)	0.64 (0.19–0.93)	0.28 (0.05–0.75)	0.93 (0.56–0.99)	0.74 (0.29–0.95)
Strike latency	8.18 (5.65–11.85)	8.21 (5.79–11.64)	10.52 (7.50–14.76)	10.55 (7.52–14.81)	5.60 (3.70–8.47)	5.61 (3.85–8.19)	7.20 (5.06–10.22)	7.22 (5.18–10.05)
Probability of a successful strike	0.16 (0.05–0.44)	0.11 (0.03–0.33)	0.22 (0.08–0.50)	0.15 (0.05–0.39)	0.15 (0.04–0.43)	0.10 (0.02–0.33)	0.21 (0.07–0.48)	0.14 (0.04–0.37)
Attack error	26.77 (18.83–34.71)	19.45 (11.93–26.98)	22.17 (15.04–29.31)	14.85 (7.20–22.50)	18.94 (9.79–28.09)	27.39 (18.46–36.31)	14.34 (6.26–22.43)	22.8 (14.89–30.68)

Values are extracted from the model of best fit (see Table 3).

**Table 3.** Effects of prey movement (erratic, linear), size (small, large), and glossiness (matte, glossy), mantid experience, and animation replicate on mantid attack behaviors.

Response variables	Explanatory variables	*χ* ^2^	df	*P*-value
Probability of tracking (*n* = 118, 17 individuals)	Glossiness	0.683	1	0.408
	**Size**	**14.917**	**1**	**<0.001**
	**Trajectory**	**7.628**	**1**	**0.006**
	**Animation replicate**	**11.152**	**2**	**0.004**
	Experience	2.740	1	0.098
Probability of striking (*n* = 52, 14 individuals)	Glossiness	2.646	1	0.104
	Size	3.702	1	0.054
	Trajectory	1.191	1	0.275
	Animation replicate	1.085	2	0.581
	Experience	3.273	1	0.070
Strike latency (*n* = 75, 13 individuals)	Glossiness	0.0003	1	0.987
	Size	2.131	1	0.144
	**Trajectory**	**5.105**	**1**	**0.024**
	Animation replicate	1.192	2	0.551
	Experience	0.010	1	0.920
Probability of a successful strike (*n* = 75, 13 individuals)	Glossiness	0.604	1	0.437
	Size	0.408	1	0.523
	Trajectory	0.028	1	0.867
	Animation replicate	5.362	2	0.068
	Experience	2.318	1	0.128
Attack error (*n* = 61, 13 individuals)	Glossiness	0.050	1	0.823
	Size	1.695	1	0.193
	Trajectory	0.009	1	0.924
	Animation replicate	3.256	2	0.196
	Experience	0.213	1	0.644
	**Glossiness: trajectory**	**5.131**	**1**	**0.024**

Interaction terms are reported if included in the model of best fit, indicated by AIC. Bold entries indicate variables with *P*-values < 0.05.

To investigate the impact of treatments on striking behavior, mantids that did not strike in response to a treatment the first time were shown the treatment again. This procedure increased the number of recorded strikes from 39 (first view) to 75 (all views). For instances when the mantid struck at the target, target trajectory significantly influenced the strike latency (*χ* = 5.11, df = 1, *P* = 0.024; Fig. 2b; Table 2). Mantids took longer to strike at targets moving in a linear trajectory than those moving in an erratic trajectory (Fig. 2b). There were no other significant effects of variables on strike latency (Tables 2 and 3).

Our treatments did not influence the probability of catching the target (probability of successful strike; Tables 2 and 3). However, there was an interactive effect of glossiness and trajectory on attack error (*χ* = 5.13, df = 1, *P* = 0.024; Fig. 2c, Table 2). For glossy targets, attack errors were larger (ie accuracy was lower) when targets followed an erratic rather than a linear trajectory. For matte targets, the reverse pattern was observed: attack errors were larger when targets followed a linear rather than an erratic trajectory. We can also interpret results based on the movement pattern. When prey moved linearly, attack errors were greater for matte targets than glossy targets, whereas when prey moved erratically, attack errors were greater for glossy than matte targets. On average, attack errors for matte-linear and glossy-erratic targets were 25 mm from the center of the target, which was 8 mm larger than for matte-erratic or glossy-linear targets. This difference could influence capture success in natural conditions.

## Discussion

Our results indicate that the physical characteristics of prey can interact with movement trajectory to impact predator hunting behavior. Praying mantids were more likely to track targets moving in a linear trajectory, and the probability of tracking was further increased for larger targets. We observed that mantids only attacked after first tracking the target. Therefore, in our experiment, reducing the probability of tracking through small size and erratic movement is the most effective strategy to avoid a mantid attack. Of those mantids that did attack, the attack errors were larger toward erratically moving glossy targets than linearly moving glossy targets. However, for matte targets mantid attack errors were lower toward erratically moving matte targets than linearly moving matte targets. This indicates that the effects of glossiness on predator attack error are influenced by movement predictability. Together, our results indicate that prey movement trajectory has the strongest influence on predator hunting behavior, but prey physical characteristics mediate these impacts.

Praying mantids were more likely to track large or linearly moving targets than small or erratically moving targets. This may represent their assessment of whether breaking camouflage to track prey is worthwhile. Predators likely adjust their behavior during predation to balance the potential energy reward against the related costs (eg energy use, reduction in prey activity; Lima and Dill 1990; Shine and Sun 2003; Griffiths 2020). Ambush predators rely on camouflage to avoid detection by prey so the prey will move close enough for the predator to attack (Pembury Smith and Ruxton 2020). Remaining stationary is important to maintain camouflage because movement is one of the most powerful visual cues to break camouflage (Regan and Beverley 1984; Rushton et al. 2007; Yin et al. 2015). Many ambush predators, including praying mantids, assess prey size and are more likely to respond to and attack larger prey (Shine and Sun 2003; Johansson et al. 2004; Prete et al. 2011, 2013; Tan et al. 2024a). Our result aligns with this previous research and suggests that mantids are more likely to engage with prey that may provide a greater energy reward. We also detected that mantids were more likely to visually track linearly moving prey, which may be because prey moving erratically are more difficult to localize and capture (Humphries and Driver 1970; Richardson et al. 2018; Tan et al. 2024b). Many insects move erratically, and our results support the idea that this movement strategy can reduce the possibility of attack from undetected predators (Humphries and Driver 1970).

Of those mantids that did attack the target, they attacked erratically moving targets more quickly than linearly moving targets. This result differs to our initial prediction that targets moving predictably would be attacked more quickly because they should be easier to localize. Instead, it may be that the erratic movement better mimics movement of prey preferred by mantids. Rainforest praying mantids eat a diverse range of insects and spiders, which they capture when the prey moves too close to the vegetation they are hiding in. Near vegetation, insects may exhibit more complex flight patterns for collision avoidance or as a search strategy to locate resources (Bell 1990; Crall et al. 2015; Singh et al. 2024). Additional field research is required to determine if praying mantids more often interact with erratically moving prey.

Mantid attack error was impacted by a combination of target trajectory and glossiness. For glossy targets, mantids made larger errors when targets were moving erratically compared with linearly. Prey may evolve a glossy appearance for a variety of functions, including non-visual functions (eg water repellence, thermoregulation; Vincent and Wegst 2004; Shi et al. 2015). Our results suggest that for glossy organisms, predation risk could be reduced by employing complex or erratic movement patterns. This aligns with previous research that discovered erratic movement can enhance the benefits of flash coloration for prey in motion (Murali and Kodandaramaiah 2020). Equally, our results suggest that for erratically moving prey, gloss may reduce predation risk: mantids made larger errors when erratic targets were glossy than when they were matte. This result supports our prediction, based on previous studies (Henríquez-Piskulich et al. 2023; Franklin et al. 2024), that gloss provides an anti-predator advantage when prey are difficult to localize. Both these previous studies used physical targets with movement paths that included variation in depth (ie distance between the target and the predator), which is more similar to our 2D erratic movement, rather than our 1D linear movement. Our results, combined with those of previous studies, suggest that the anti-predator benefits of glossiness for moving prey may depend on prey movement patterns (Henríquez-Piskulich et al. 2023; Franklin et al. 2024).

Counterintuitively, we also detected that mantids made larger attack errors toward matte targets moving linearly. We expected linear-matte targets to be the easiest target to capture because they are less visually changeable than glossy targets and their future position should be easier to predict than erratically moving targets. It is possible, however, that matte targets with 1D linear movement provided reduced motion cues compared with other target treatments. Motion can be detected by first-order and second-order motion cues. First-order motion involves correlated changes in luminance over space and time, such as a dark object moving across a lighter background. In our experiment, both linear and erratic trajectories produce first-order motion, but the erratic movement may enhance first-order motion signals. This is because during upward or downward movement, which is only present in the erratic trajectory, the leading edge is the long axis of the ellipse, producing more extensive edge motion. First-order motion is an important component of target tracking for predatory insects (O'Carroll 1993; Kral and Prete 2004; Geurten et al. 2007), and perhaps erratic movement provides more salient cues. The targets we created also produced second-order motion cues, particularly the glossy targets. Second-order motion is the perception of motion not by luminance cues, but by other properties such as texture or flicker. Both targets were textured, but this texturing only produced large changes in reflectance with movement for the glossy target (ie flashes or shifting reflections on the surface). This provides second-order motion cues, which mantids can use to target prey (Nityananda et al. 2019). In addition, glossy targets have higher internal contrast because they are not uniform, which also likely increases contrast with the background. This may contribute to the observed result that mantids had lower attack errors toward linear-glossy targets than linear-matte targets. However, further research is required to disentangle these possibilities or identify other drivers of this unexpected result.

Whilst our results aligned with previous research investigating anti-predator benefits of glossiness for moving prey, it is important to highlight the limitations of presenting animated glossy targets versus physical glossy targets. Physical objects create glossy reflections that are in a slightly different position for each eye of the viewer (Chadwick and Kentridge 2015), but animations cannot replicate this unless 3D glasses are used (Nityananda et al. 2016b). This disparity is important for humans to be able to distinguish glossy reflections from differently colored patches on a surface, and to judge the degree of glossiness (Obein et al. 2004; Chadwick and Kentridge 2015). Furthermore, the disparity can make the reflections look like they are floating above or below the surface, which may disrupt depth cues (Chadwick and Kentridge 2015; Nityananda and Read 2017; Adams et al. 2019). Whilst previous research with jumping spiders suggests that the impact of glossiness on attack error is not due to disruptions of depth cues (Franklin et al. 2024), it is possible that binocular disparity may impact localization. Additionally, physical targets in direct light can produce very bright glossy reflections relative to the rest of the visual scene, whereas a monitor will have a smaller intensity range. It is not known whether the intensity of the glossy flash is important for disrupting predator attacks. Previous research investigating flash coloration or motion dazzle markings (ie patterns, such as stripes, that may interfere with predator capture of moving prey) often have not found strong support for an influence of contrast on predator attack behaviors (Hogan et al. 2016; Murali 2018; Kodandaramaiah et al. 2019); however, these studies were also conducted using computer animations. Since we did detect an impact of glossiness on predator attack error, it is likely that the characteristics the animations can portray—changeability, flashiness, spatial patterns of reflectance—contribute to the impacts on predator behaviors. However, additional research is required to pinpoint the specific characteristics of glossy animals that impact predator attack behaviors.

Our video animation replicates also influenced whether praying mantids would track the target. For 1 animation replicate (replicate c), mantids were much less likely to track the target than the other 2 animation replicates. These results may be due to characteristics of the target or the movement path. The target color was based on the background image uploaded to the animation software. The different images used resulted in 2 targets reflecting mostly white light off the dorsal surface and 1 target reflecting mostly blue light off the dorsal surface. This would impact contrast, with the targets reflecting white light contrasting more against the gray background than the target reflecting blue light. The bluer targets were less likely to be tracked, which may be because mantids prefer to track high contrast targets (Kral and Prete 2004; Prete et al. 2011). The movement paths also differed among replicates. Each path was generated from real insect flight parameters, with variation incorporated into these parameter estimates. This resulted in differences among paths in where the target would rise or fall and the degree of angular change in movement. Prey position in the visual field and movement trajectory can influence mantid attack behaviors, with some species preferring to attack prey in the lower center visual field and those moving downwards (Kral and Prete 2004). Therefore, it is possible that characteristics of the erratic movement paths influenced whether mantids would track the targets, but further investigation is required to determine what characteristics may have influenced this response.

Previous research with praying mantids has discovered that response to a stimulus can change throughout an experiment with additional experience (Maldonado 1972; Henríquez-Piskulich et al. 2023), however we did not detect an improvement through repeated exposure to the animations. This may be because erratic movements are difficult to predict, which could limit the ability of predators to learn and improve (Tan et al. 2024b). Half of the treatments involved erratic movement, and we include 3 different movement path replicates for the erratic treatments. This unpredictability may have limited the mantids’ ability to improve their attack strategy, which could be an additional benefit for erratically moving prey.

Together, our results indicate that prey physical characteristics interact with movement trajectory to influence predator attack behaviors. Some prey characteristics, such as glossiness, may only provide anti-predator benefits when associated with fast or erratic movement (Henríquez-Piskulich et al. 2023). This aligns with previous studies that have found benefits or costs of prey coloration depend on speed and trajectory (Stevens et al. 2008; Scott-Samuel et al. 2011; Umeton et al. 2019; Murali and Kodandaramaiah 2020; Henríquez-Piskulich et al. 2023; Silvasti et al. 2024). For example, flash coloration may be beneficial against avian predators for fast-moving prey, but detrimental for slow-moving prey (Silvasti et al. 2024). Only a few studies have investigated these effects with animal predators, and we generally have a poor understanding of the perceptual mechanisms behind these impacts. Filling this gap will help us understand whether the costs and benefits of anti-predator strategies in motion are specific to certain predators and to uncover how natural selection may influence prey appearance and movement.

## Supplementary Material

araf107_Supplementary_Data

## Data Availability

Analyses reported in this article can be reproduced using the data and code provided by Wang et al. (2025) .
