# Supplementary material for: Prey movement, size, and glossiness interact to impact praying mantid attack behaviors
Source: Behav Ecol. 2025 Sep 10;36(5):araf107. doi: 10.1093/beheco/araf107 (PMC12527264; doi:10.1093/beheco/araf107)
Supplement: araf107_Supplementary_Data [file araf107_supplementary_data.docx]

**Supplementary Material**

**Prey movement, size and glossiness interact to impact praying mantid attack behaviours**

Yvonne Wang^1^, Devi Stuart-Fox^1^, Patricia Henriquez-Piskulich^1^, Amanda M. Franklin^1,2^

**Affiliations**:

1 School of BioSciences, The University of Melbourne, Parkville, Vic., Australia

2 Department of Ecological, Plant and Animal Sciences, La Trobe University, Bundoora, Vic. Australia

* Correspondence: Amanda Franklin. email: a.franklin@latrobe.edu.au


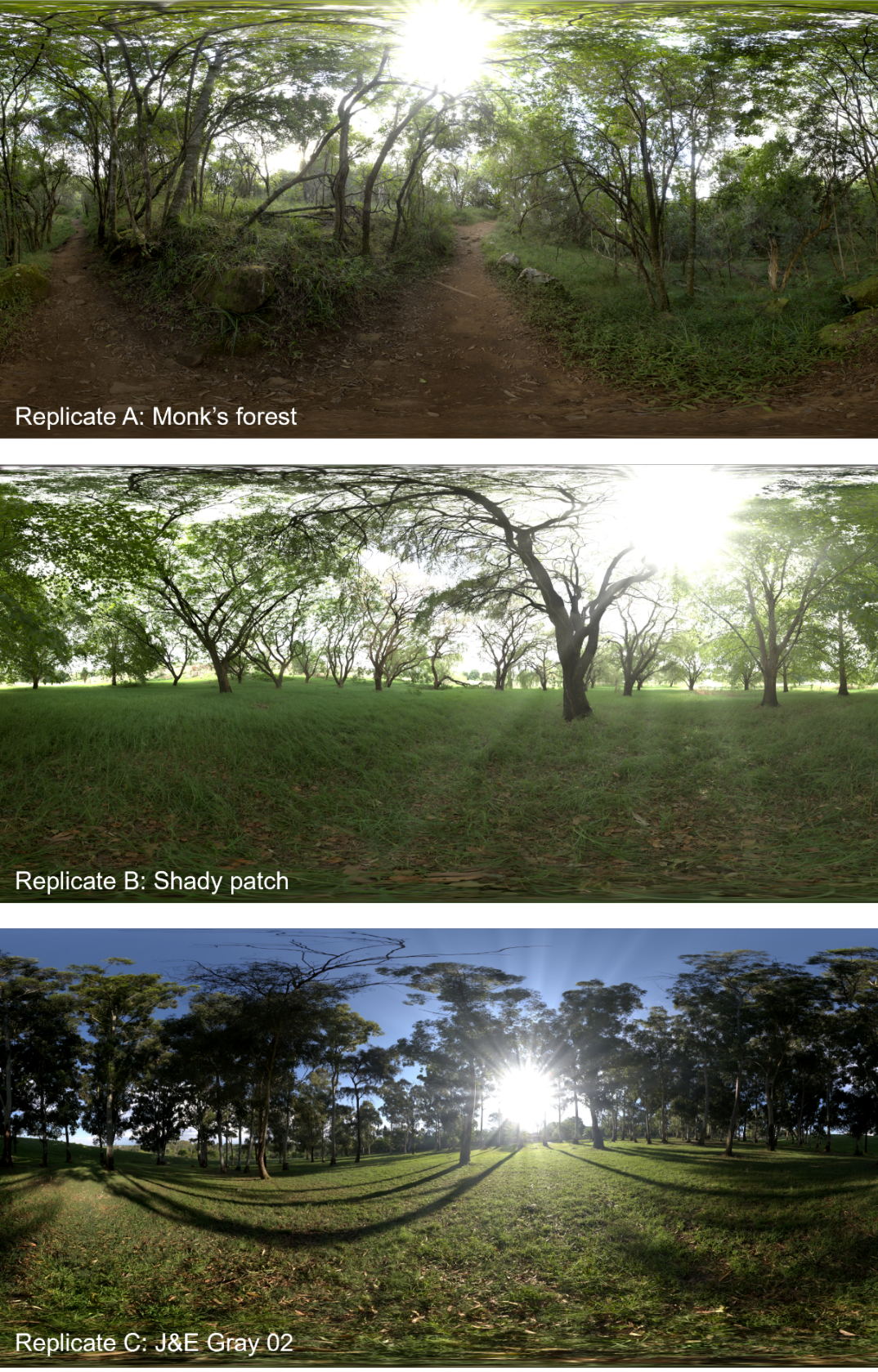


Figure S1: 360° images of natural forest scenes downloaded from PolyHaven ([www.PolyHaven.com](http://www.PolyHaven.com)). These images were imported into Maya to created the lighting environment for three different animation replicates. These images impact the reflections off the glossy targets and the average colour used to create the matte target. Name of each image on PolyHaven indicated in bottom left corner.


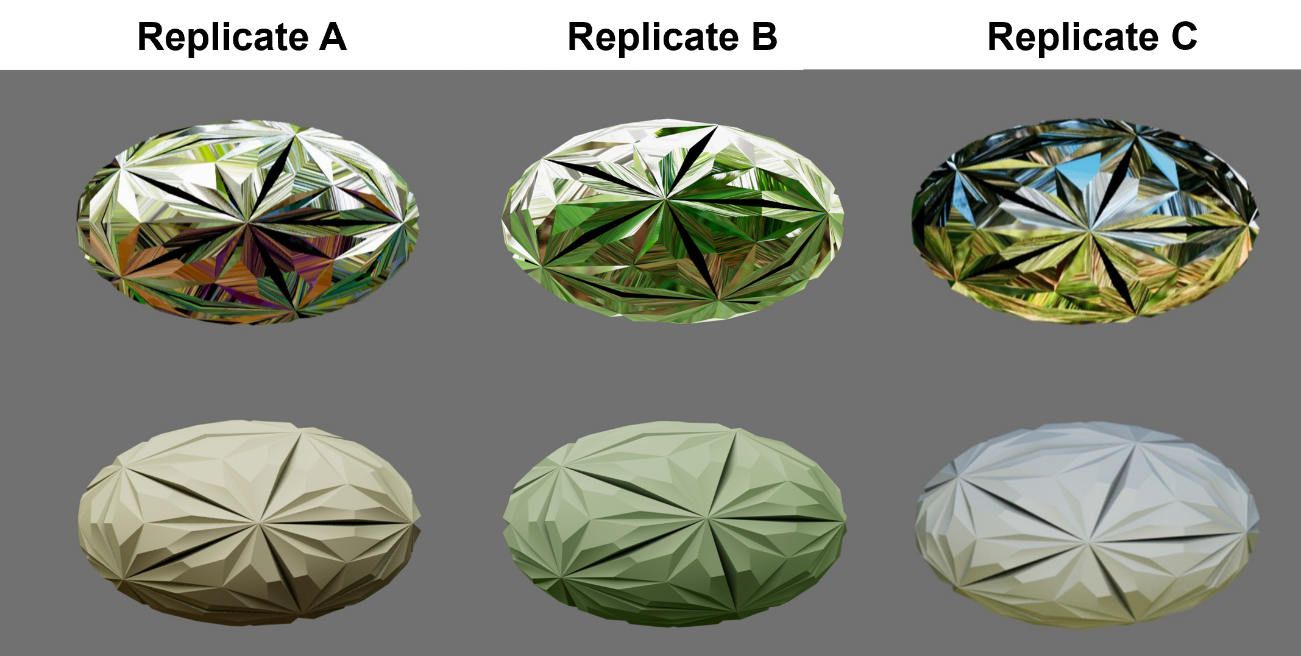


Figure S2: Glossy and matte targets produced using the three 360° background images depicted in figure S1. Matte targets are the average RGB values of the glossy target. All targets show shading because the light is modelling to come from above (i.e. the sky and sun).


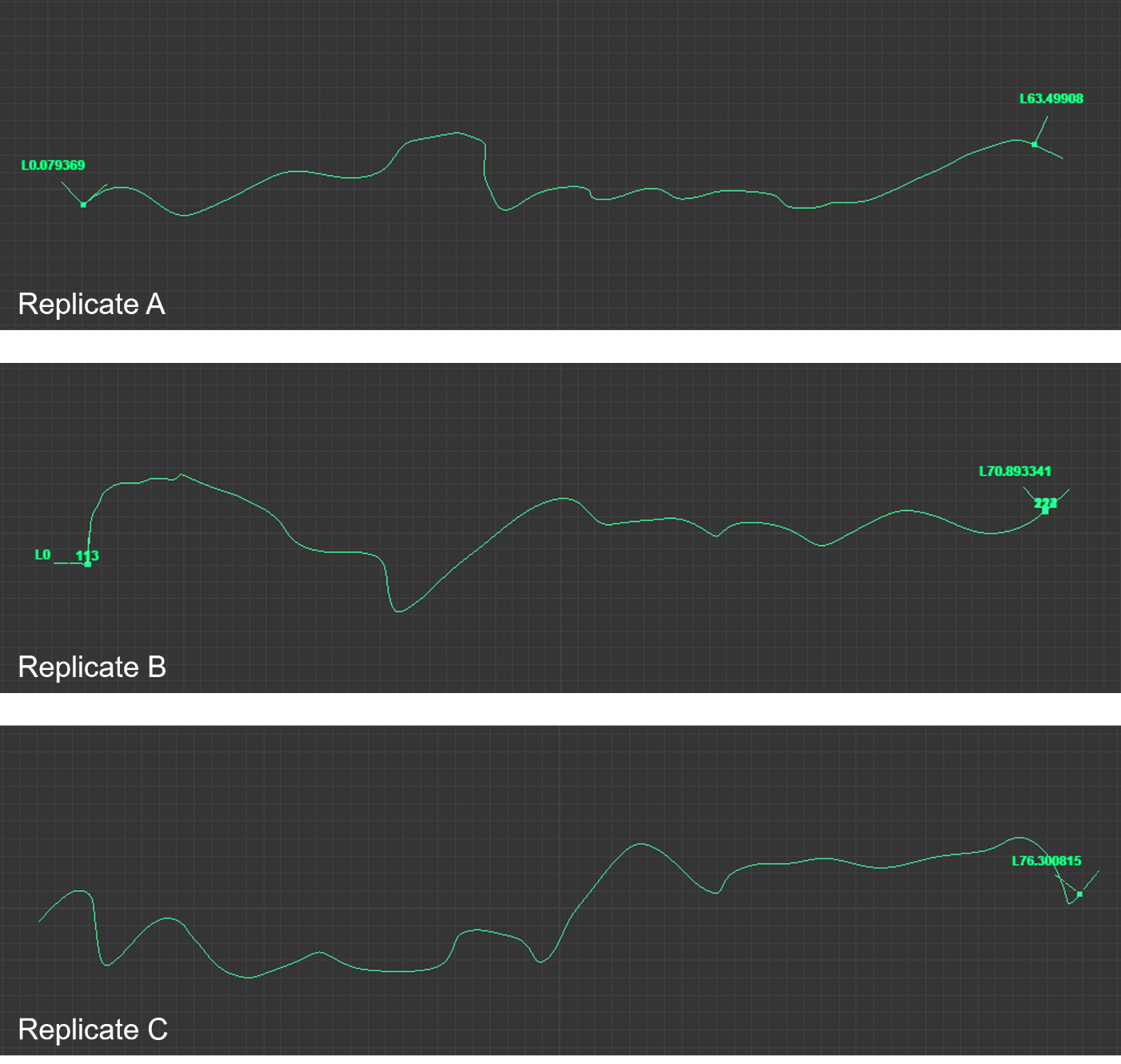


Figure S3: Movement paths generated using trajr package in R and housefly movement parameters.
